# Fatty acid and mineral contents of *Lycium ruthenicum* Murr. and antioxidant activity against isoproterenol‐induced acute myocardial ischemia in mice

**DOI:** 10.1002/fsn3.1393

**Published:** 2020-01-07

**Authors:** Irma Belinda Yossa Nzeuwa, Hui Xia, Yuanyuan Shi, Chao Yang, Muhammad Waseem Shah, Baofu Guo, Liya Wang, Guiju Sun

**Affiliations:** ^1^ Department of Nutrition and Food Hygiene Key Laboratory of Environmental Medicine Engineering of Ministry of Education School of Public Health Southeast University Nanjing China; ^2^ Key Laboratory of Drug Quality Control and Pharmacovigilance Ministry of Education China Pharmaceutical University Nanjing China; ^3^ Nanjing Municipal Center for Disease Control and Prevention Nanjing China

**Keywords:** antioxidant activity, fatty acid content, isoproterenol, *Lycium ruthenicum* Murr., minerals content, myocardial ischemia

## Abstract

Dried fruits of black goji were investigated for their fatty acid, mineral contents, and antioxidant activity against isoproterenol‐induced acute myocardial ischemia in mice was revealed. It was observed that the key fatty acids from *Lycium ruthenicum* Murr. (LRM) oil studied included linoleic (59.38%), oleic (20.85%), palmitic (7.07%), linolenic (2.98%), and stearic acids (5.31%), which together comprised 95.59% of the total fatty acids. The key mineral nutrients studied were potassium (17,631.15 mg/kg), calcium (2004.4 mg/kg), and magnesium (1,274.6 mg/kg), while copper, iron, manganese, and zinc were found in trace. Moreover, oral administration of water extraction of LRM exhibited significant reduction of enzyme activities, and MDA level triggered by ISO to be near normal level, while exhibited a significant increase of SOD and GSH activities. Our results provide deep insight on LRM as a potential source of high‐value phytochemicals for the development of new functional food and beverage products.

## Graphical abstract

Lycium ruthenicum nutrient content not determined yet including mineral and fatty acid was investigated. The whole crude water extract was also investigated for its antioxidant activity against acute myocardial ischemia in mice.

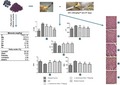

## 1 INTRODUCTION

Berry fruits have attracted an overwhelming research focus within the past years because of their abundant source of natural antioxidants essential to human, varied metabolites with interesting biological activities and for their nutritional value. From the evidence in the literature, their use have been strongly associated with reduced risk of chronic diseases, including cardiovascular disease, cancer, diabetes, Alzheimer's disease, in addition to other health benefit (Zhang & Hamauzu, 2004). Dried fruits of *Lycium* plants account for approximately 80 species in the world (Levin & Miller, 2005) are very popular berries in Asia and other countries (Seeram, 2008). In recent years, the genus *Lycium* has especially received considerable attention (Kulczynski & Gramza‐Michalowska, 2016; Wang et al., 2018). Also referred to wolfberry or Fructus lycii, *Lycium* fruits the genus *Lycium* has 3 main species (*L. barbarum L*.,* L. ruthenicum* Murray, and *L. chinense* Miller) which have been used as traditional medicinal foods in China and many other countries for centuries (Zhao et al., 2018). *Lycium ruthenicum Murr*. fruits (LRM, “Hei Guo Gou Gi” in Chinese or called “black goji berry”) (Xin, Zhu, Du, & Xu, 2017) are known to be a good source of functional components including polysaccharides, phenolic compounds, anthocyanins, fatty acids, and minerals (Peng et al., 2012). Among these chemical constituents, anthocyanins and polysaccharides have been proven to be the major bioactive compounds (Qian, Zhao, Yang, & Huang, 2017), with anthocyanins, the main functional compositions in these berries (Islam, Yu, Badwal, & Xu, 2017; Zheng et al., 2011). LRM has been used for the treatment of many diseases, recent pharmacological studies reported a lot of biological activities including, anti‐cancer, anti‐diabetes (Nzeuwa, Xia et al. 2017), anti‐fatigue, and antioxidant activities (Islam, Yu et al. 2017). Qinghai was reported to be one of the most important regions, considered as the “standard region” for LRM production in China due to higher quality level (Zheng et al., 2011). LRM seeds account for at least 30% of the total dried fruits (Zhao et al., 2018) with growing demand on nutritional and functional properties of seed oils, the chemical composition and biological activities of nonconventional seed oils are concerned more and more. There is an exhaustive amount of reports on the medicinal properties of LRM fruits. However, investigations regarding LRM seed oil and anthocyanins are still poorly documented, and there is no study that has attempted in characterizing their mineral components or evidence of their uses against ISO‐induced myocardial ischemia. The daily intake of mineral nutrients is, by nature, small, especially when compared with macro nutrients such as carbohydrates. Since minerals are indispensable to functioning of the organism, they must be present in our diet.

In the course of our continuing search for metabolites of biological importance from LRM, as LRM were widely used as fruits and tea for the daily supplementation, we investigated fatty acid and minerals contents of the fruits and its water extractions. Furthermore, the antioxidant activity of the fruits used in traditional medicine and in daily life (tea, dry fruits) prompted us to investigate the in vivo cardioprotective effects in detail; therefore, we designed an in vitro study aimed at evaluating the cardioprotective effect of LRM on ISO‐induced biochemical and histopathological changes in mice.

## 2 MATERIALS AND METHODS

### 2.1 Plant material

Black goji berries (LRM) were obtained from Qinghai province, precisely Ge Er Mu in September 2017. The fully ripened fruits were hand‐picked and dried, then blended to a fine powder using an XS‐10 pulverizer (Zhaoshen Technology Co., Ltd.) and sieved through a 40‐mesh sieve.

### 2.2 Drug and Chemical reagents

Petroleum ether, methanol, nitric acid (HNO_3_), potassium chloride, and sodium acetate were from Sinopharm Chemical Reagent Co. Ltd. Water was purified by using a Milli‐Qplus system from Millipore; nylon filters (0.45 μm, 0.22 μmpore size) were from Nanjing Mabel Biotechnology Co., Ltd.

Isoproterenol hydrochloride and propranolol were from Shanghai pharmaceuticals Co. Ltd. Lactate dehydrogenase (LDH), creatine kinase (CK), superoxide dismutase (SOD), and malon dialdehyde (MDA) assay kits were produced by the Institute of Nanjing Jiancheng Biology Engineering. All chemicals and reagents used in this study were of analytical grade from Dikma Technologies Inc. Distilled deionized water and ultrahigh purity commercial acids were used to prepare all reagents, standards, and samples.

### 2.3 Optimized extraction of oil from the Dried Fruits

Crude oil was extracted according to the method described by Nehdi, Sbihi, Tan, & Al‐Resayes, (2013) with a slight modification using a Soxhlet apparatus with petroleum ether for 2 hr. Oil extracted was weighed, and oil content of the fruits was expressed as percent of dry matter mass. The main fatty acid content from LRM including, palmitic acid, linoleic acid, linolenic acid, stearic acid, and oleic acid were analyzed by GCMS using an Agilent 7890A gas‐liquid chromatograph equipped with a dual flame ionization detector and an HP‐88 column (60 m 0.25 mm, film thickness 0.25 m; Agilent Technology). Each of the experiments was repeated three times. Results were expressed as relative percentages.

### 2.4 Mineral content assay

Mineral contents including potassium, calcium, magnesium, copper, zinc, manganese, and iron were measured using the standard protocol used by Kumari, Parida, Rangani, & Panda, (2017) with slight modification as described in our previous study (Nzeuwa et al., 2019): LRM powder was first dried at 70°C in an oven until reached a constant weight. After drying, approximately 0.5 g of the dried powder was digested with a mixture of 5 ml of 65% HNO_3_ and 2 ml of 35% hydrogen peroxide (H_2_O_2_), using a 25 ml volumetric flask. When a colorless liquid appeared indicating the completion of digestion, volume of the digested sample was completed to 25 ml with ultradeionized water, and mineral concentrations were determined. After digestion treatment, sample was filtrated through Whatman Ashless No 42 paper filter. Filtrate was collected and content of various ions was determined. Mineral content of the LRM sample was quantified against standard solutions of known concentrations, which were analyzed simultaneously. Results were expressed as mg/kg edible DW.

### 2.5 Water extraction procedure

LRM powder was extracted using the method described by Peng et al., (2012), slightly modified. In brief, LRM powder was extracted three times with water (1:60 *w/v*) at 40°C by ultrasonication for three hours. The extract obtained was filtrated through Whatman duplex qualitative filter from General Electric Biological Technology, freeze‐dried, and blended. The powder obtained was sieved and stored at 4°C for further analysis.

### 2.6 Experimental animals

Forty ICR mice weighing 21 ± 1 g were used in this study. The animals were housed in clean cages and were maintained under standard laboratory conditions: air‐conditioned room kept under standard conditions of humidity (50 ± 5) %, temperature (25 ± 2) °C, and light (alternate 12 hr light/12 hr dark cycle). The bedding material of the cages was changed every two days. They were allowed free access to standard laboratory diet and water ad libitum during the experimental period. All procedures were in accordance with the National Institute of Health's guidelines regarding the principles of animal care and approved by the institutional animal care guidelines. The experimental protocol was approved by the China pharmaceutical university Institutional Animal Ethics Committee (N#201608341).

#### 2.6.1 Experimental design

LRM water extract powder was suspended in 0.9% saline solution and each animal belonging to different groups except the control group received *L. ruthenicum* Murr. Suspension at a dose of 750 and 375 mg/kg body weight everyday respectively by intragastric intubation.

A total of 40 mice were randomly allocated into five groups of eight animals each. Mice in groups I (normal group) and II (negative control) received 1.0 ml of 0.5% saline solution every day via intragastric intubation. Groups III and IV received LRM suspension via intragastric intubation at a daily dose of 750 and 375 mg/kg body weight respectively for a period of 8 days; group V mice received propranolol (15 mg/kg) for 8 days then on seventh and eighth days mice from groups II, III, and IV received isoproterenol (20 mg/kg body weight) injections (i.p) twice at an interval of 24 hr. All of these doses were converted according to the clinical dose. At the end of the experimental protocol, blood was collected through orbital plexus and serum separated for different biochemical investigations.

#### 2.6.2 Determination of the MDA and GSH levels and LDH, CK, SOD activities

Animals were sacrificed by cervical decapitation directly after blood collection, and hearts were dissected out immediately, weighed, excised, and washed with ice‐cold saline. 10% (w/v) homogenates in phosphate buffer (50 mM, pH7.4) were prepared. A small portion of the homogenate was used for the estimation of MDA, the other portion of the homogenates was centrifuged at 7,000 rpm in 4°C for 10 min using high‐speed cooling centrifuge. The clear supernatant was used for the estimation of reduced glutathione (GSH) and superoxide dismutase (SOD).

#### 2.6.3 Histological studies

Heart tissues from mice were fixed using 10% buffered formalin and embedded in paraffin. Thereafter, sections were cut into 5 μm and stained using hematoxylin and eosin (HE). These sections were then examined under a light microscope to examine the architecture of the myocardium.

### 2.7 Statistical analysis

Analytical analyses were conducted in triplicate, and all of the data were expressed as mean of three replicates. The statistical analysis was performed using Microsoft Excel 2010 (Microsoft Corp.).

Results regarding animal experiment were expressed as the mean ± *SEM* (standard error of mean) and analyzed by one‐way ANOVA with GraphPad Prism 7.0 software as described in Dunnett's test. The probability (P) value less than .05 was considered to be statistically significant. *p < .05, **p < .01 considered very significant and ***p < .001 considered highly significant, when compared with ISO‐positive control group.

## 3 RESULTS AND DISCUSSION

We investigated LRM in terms of macro‐ and micronutrients content. Nutrients chosen in this study were found to have highly significant nutritive values and pharmacological properties. In addition to that they are indispensable to the maintenance of life.

### 3.1 Optimized Extraction of oil from the Dried Fruits

Fatty acids (FAs) are very important for human diet and disease prevention. Among these, PUFAs are of great relevance because they can prevent cardiovascular‐related diseases (Cheng, Zhu, Hamaker, Zhang, & Campanella, 2019). As shown in Table 1, the main fatty acid composition of LRM included linoleic acid with the highest value (59.38%), oleic acid (20.85%), palmitic acid (7.07%), linolenic acid with the lowest value (2.98%), and stearic acid (5.31%), which together comprised 95.59% of the total fatty acids. Linoleic acid has been reported for its hypocholesterolemic ability, its effect to increase membrane fluidity, and to lower blood pressure (Chan, Bruce, & McDonald, 1991). Linoleic acid, known to be an essential fatty acid (EFAs), has recently gained much attention due to a series of pharmacological activities, which include hypolipidemic, antiaggregating, antioxidant, antithrombosis, antiglycemic, and anticancerous activities (Bhatia, 2015). Therefore, LRM oil can be used as dietetic oil (edible cooking oil, salad oil, and frying oil) and for diverse functional foods and cosmetic applications such as for the manufacture of margarine and can be incorporated into ice cream products. These results were different from those reported by Zhao et al., (2018), in which fatty acid and phytosterol composition, and the biological activities of *Lycium ruthenicum* Murr. seed oil was investigated. The report showed a higher value of PUFAs than ours (74.58% for linoleic and 6.6% for linolenic acid), while MUFAs and SFAs were at least two times lower. In addition, Chi, Xiao, Dong, qin Yang, & Hu, (2016) reported lower values of linoleic, oleic, and stearic acids contents (54.11%, 20.7% and 3.56%, respectively) and higher values of palmitic (12.49%) and linolenic (4.99%) acid contents than ours. Our report was somehow similar with those reported by Nehdi et al., (2013) in which *Citrus colocynthis* (L.) shared seed oil and Helianthus annuus (sunflower) seed oil were evaluated characterized and compared with a slight difference observed in palmitic (9.74% and 6.32%, respectively) and stearic contents (7.37% and 4.66, respectively). Our results show that LRM oil can be used as dietetic oil and for diverse food applications as sunflower seed oil.

**Table 1:** Minerals and fatty acid content of *Lycium ruthenicum* Murr

Minerals (mg/kg)	
K	
Mg	1,274.6
Ca	2004.4
Cu	7.84
Zn	15.86
Mn	12.19
Fe	8.18
Fatty acids (%)	
Linoleic	59.38
Oleic	20.85
Palmitic	7.07
Linolenic	2.98
Stearic	5.31

### 3.2 Mineral Content

Minerals are one of the four groups of essential nutrients essential to the maintenance of life. Mineral elements from LRM expressed in mg/kg of dry weight (DW) are summarized in Table 1. From the results, we clearly observed that the predominant mineral nutrients included potassium (K) with a value of 17,631.15, followed by calcium (Ca, 2004.4) and magnesium (Mg, 1,274.6). They represent the major minerals in the human body, known as "trace elements," but having a specific biochemical functions. Copper (Cu), iron (Fe), manganese (Mn), and zinc (Zn) were also reported with content value of 7.84, 8.18, 12.19, and 15.86 mg/kg, respectively. All mineral nutrients in LRM fruits were relatively higher than red goji berries reported in our previous study (Nzeuwa et al., 2019), except for the content of copper. The difference in soil, the harvest time, and the fruit genus may hold clue to the explanation of the difference observed.

### 3.3 Effect of LRM pretreatment on serum myocardial marker enzymes on mice

**Figure 1:**
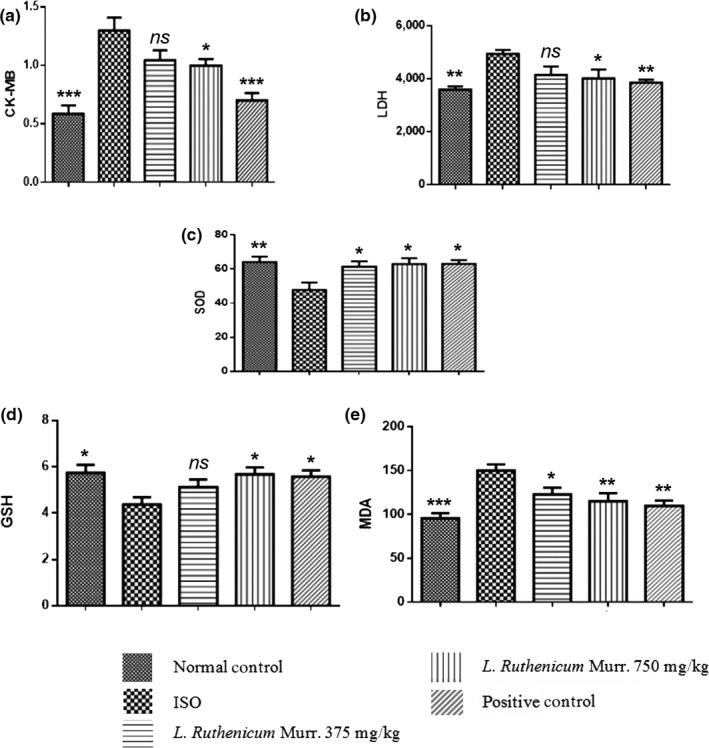
Effect of *Lycium ruthenicum* Murr. pretreatment on serum myocardial marker enzymes, lipid peroxidation and antioxidants of experimental mice

Table 2 exhibits the changes in CK‐MB (Figure 1a) and LDH (Figure 1b) in serum. In ISO‐alone‐treated animals, all parameters significantly increased compared with control group (*p* < .001), a decrease was noticed in LRM pretreated ISO‐induced animals (*p* < .05) as compared to the respective value of ISO‐induced animals. Compared with the ISO group nonsignificant fall of LDH and CK‐MB in serum was seen in the LRM with low dosage (375 mg/kg) while in LRM high‐dosage pretreatment group (750 mg/kg) the activity of CK‐MB and LDH decreased significantly (*p* < .05), suggesting a protective effect of LRM against cardiac damage due to ISO comparing to the low‐dosage pretreatment with an amelioration of the myocardial histopathology. Our results were in accordance with those reported by Hou, Chen, Shu, Feng, & Hung, (2019) in which the protective effect of supplementation with *Lycium ruthenicum* Murray extract from exhaustive exercise‐induced cardiac injury in rats was investigated.

**Table 2:** Effect of *Lycium ruthenicum* Murr. on serum myocardial marker enzymes of experimental animals

Groups	CK‐MB(U/mL)	LDH(U/L)
Normal saline (1 ml)	0.585 ± 0.072*	3,592.814 ± 121.842**
Isoproterenol (20 mg/kg)	1.297 ± 0.110	4,944.111 ± 143.488
*L. ruthenicum*(375 mg/kg)	1.044 ± 0.084*ns*	4,149.700 ± 315.190*ns*
*L. ruthenicum* (750 mg/kg)	0.998 ± 0.055***	4,013.972 ± 333.686***
Propanolol (15 mg/kg)	0.700 ± 0.061*	3,856.287 ± 118.054**

* Statistically significant (*p*<.05) as compared to ISO group.

** Very significant (*p*<.01) as compared to ISO group.

*** Highly significant (*p*<.001) as compared to ISO group.

^ns^ Non significant.

ISO‐treated experimental mice exhibited a significant heart injury as evidence by a significant elevation in the biochemical markers of cardiac function in comparison to those of the normal healthy group. However, oral administration of LRM exhibited dose‐dependent significant reduction of the increased enzyme activities triggered by ISO to near normal levels. Obviously, LRM has restored all the biochemical parameter levels significantly to near normal levels.

### 3.4 Effect of *L. ruthenicum* Murr. Pretreatment on Lipid peroxidation and antioxidants

Compared to the respective values of normal group, in ISO‐treated animals we observed a significant (*p* < .001 and .05) decrease in the activities of SOD (Figure 1c), and the level of GSH (Figure 1d). Pretreatment with LRM (750 mg/kg) significantly (*p* < .05 or *p* < .01) increased the activities of these antioxidants in ISO‐induced mice (Table 3). The impact on enzymic antioxidants and lipid peroxidation is indicated in Table 3. After the injection of ISO, MDA level increased significantly (Figure 1e), while SOD and GSH activity diminished in the ISO‐induced group (*p* < .001 and *p* < .01, respectively) when comparing to the normal group. This implies that animals of the ISO group exhibited an oxidative‐induced myocardial tissue injury. LRM administrated at high‐dosage substantially increase GSH activity (*p* < .05). This showed a reversed effect as compared to the ISO‐induced group.

**Table 3:** Effect of *Lycium ruthenicum* Murr. on lipid peroxidation and antioxidant in isoproterenol‐induced myocardial ischemia

Groups	MDA(nmol/g)	SOD(U/mg)	GSH(mg/g)
Normal saline (1 ml)	95.662 ± 5.730*	64.230 ± 2.830**	5.751 ± 0.331***
Isoproterenol (20 mg/kg)	150.049 ± 6.958	47.745 ± 3.969	4.376 ± 0.309
*L. ruthenicum *(375 mg/kg)	122.731 ± 7.797***	61.420 ± 2.846***	5.120 ± 0.300*ns*
*L. ruthenicum* (750 mg/kg)	115.033 ± 9.257**	62.955 ± 3.175***	5.678 ± 0.269***
Propanolol (15 mg/kg)	109.817 ± 5.998**	63.097 ± 1.999***	5.583 ± 0.099***

* Statistically significant as compared to ISO group (*p*<.05).

** Very significant as compared to ISO group (*p*<.01 ).

*** Highly significant as compared to ISO group (*p*<.001).

^ns^ Non significant.

### 3.5 Histopathological and ultrastructural alterations

**Figure 2:**
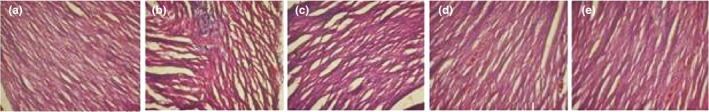
Photomicrograph of heart from mice showing cardiac myocytes. (a) Control: Photomicrograph of heart of control animal, (b) ISO‐induced mice, (c and d) Photomicrograph of heart from mice pretreataed with low and high dosage of *Lycium ruthenicum* Murr. respectively, e: positive control

In histopathological examinations showed in Figure 2, normal architecture was observed in the heart of control mice without necrosis, edema, or inflammation (Figure 2a). ISO‐induced mice (negative control) showed infracted heart with serious myocardial damage, demonstrated by irregular swelling of myocardial fiber, increased necrosis and fusion area, large gap between muscle fibers, poor structure of myocardium, and large number of inflammatory cells infiltrating the myocardial tissue (Figure 2b). This showed that myocardial fiber necrosis has occurred. The two doses of LRM treatment groups showed improved myocardial tissue damage compared with the ISO group. The myocardial tissue of high‐dosage LRM‐treated mice showed a significant reduction in the infiltration of inflammatory cells and trace or no sign of cell edema and necrosis (Figure 2d). Even though there was mild edema, the cardiac fibers were within the normal limits in the myocardium. The myocardial tissue of low‐dosage LRM*‐*treated mice showed a certain myocardial protection effect, as compared to negative control pretreatment (Figure 2c). The positive control group showed reduced inflammatory cell infiltration, local cellular edema, and occasional individual cell necrosis (Figure 2e). Results of the present investigation clearly revealed the cardioprotective effects of LRM in mice model of experimentally induced myocardial necrosis. Treatments were effective with the structure of cardiac tissue closely identical to the positive control. Pretreatment with LRM could protect the heart from ISO‐induced cardiac damage by reducing the fibers necrosis, the number of inflammatory cells and myocardial tissue to the normal level.

## 4 CONCLUSION

In summary, we investigated the fatty acid and mineral contents together with the protective effects against ISO‐induced myocardial oxidative damage of *L. ruthenicum* Murr. Our results indicated that the main fatty acid found in LRM is linoleic acid a value of 59.38%, LRM is also a source of minerals including potassium, calcium, magnesium, copper, zinc, manganese, and iron *L. ruthenicum* Murr. significantly restored myocardial damage induced by ISO in mice. *L. ruthenicum* Murr. exerted its beneficial effects on myocardial injury mainly by inhibiting oxidative damage caused by stress and by maintaining the functional integrity of myocardial tissue. These findings suggest the potential of LRM extract to serve as functional food ingredients.

## CONFLICT OF INTEREST

The authors declare that they do not have any conflict of interest.

## ETHICAL STATEMENT

Ethical Review protocol and procedures employed were ethically reviewed and approved by the China pharmaceutical university Institutional Animal Ethics Committee (N#201608341). Experiments were performed in accordance with National Institute of Health's guidelines regarding the principles of animal care and approved by the institutional animal care guidelines.

## INFORMED CONSENT

Written informed consent was obtained from all study participants.

## References

[fsn31393-bib-0001] Bhatia, A. Sharma, A. , Balgir, P. P. , Kapoor, D. & Deol, M. K. (2015). Evaluation of immunomodulatory, antioxidant and anticancerous potential of 9Z, 11E isomer of clA. International Journal of Pharmaceutical Research and Bio‐Science, 4(2), 14.

[fsn31393-bib-0002] Chan, J. K. , Bruce, V. M. , & McDonald, B. E. (1991). Dietary alpha‐linolenic acid is as effective as oleic‐acid and linoleic‐acid in lowering blood cholesterol in normolipidemic men. American Journal of Clinical Nutrition, 53(5), 1230–1234. 1673589 10.1093/ajcn/53.5.1230

[fsn31393-bib-0003] Cheng, L. , Zhu, X. , Hamaker, B. R. , Zhang, H. , & Campanella, O. H. (2019). Complexation process of amylose under different concentrations of linoleic acid using molecular dynamics simulation. Carbohydrate Polymers, 216, 157–166. 10.1016/j.carbpol.2019.04.013 31047052

[fsn31393-bib-0004] Chi, X. , Xiao, Y. , Dong, Q. , qin Yang, Y. , & Hu, F. et al (2016). Fatty acid composition of lycium ruthenicum collected from the Qinghai‐Tibetan plateau. Chemistry of Natural Compounds, 52(4), 674–675. 10.1007/s10600-016-1737-x

[fsn31393-bib-0005] Hou, C.‐W. , Chen, I.‐C. , Shu, F.‐R. , Feng, C.‐H. , & Hung, C.‐T. (2019). Protective effect of supplementation with Lycium ruthenicum Murray extract from exhaustive exercise‐induced cardiac injury in rats. Chinese Medical Journal, 132(8), 1005–1006. 10.1097/CM9.0000000000000185 30958451 PMC6595767

[fsn31393-bib-0006] Islam, T. , Yu, X. , Badwal, T. S. , & Xu, B. (2017). Comparative studies on phenolic profiles, antioxidant capacities and carotenoid contents of red goji berry (Lycium barbarum) and black goji berry (Lycium ruthenicum). Chemistry Central Journal, 11(1), 59 10.1186/s13065-017-0287-z 29086843 PMC5483215

[fsn31393-bib-0007] Kulczynski, B. , & Gramza‐Michalowska, A. (2016). Goji Berry (Lycium barbarum): Composition and Health Effects – a Review. Polish Journal of Food and Nutrition Sciences, 66(2), 67–75. 10.1515/pjfns-2015-0040

[fsn31393-bib-0008] Kumari, A. , Parida, A. K. , Rangani, J. , & Panda, A. (2017). Antioxidant activities, metabolic profiling, proximate analysis, mineral nutrient composition of *Salvadora persica* fruit unravel a potential functional food and a natural source of pharmaceuticals. Frontiers in Pharmacology, 8, 61. 28261096 10.3389/fphar.2017.00061 PMC5306401

[fsn31393-bib-0009] Levin, R. A. , & Miller, J. S. (2005). Relationships within tribe Lycieae (Solanaceae): Paraphyly of Lycium and multiple origins of gender dimorphism. American Journal of Botany, 92(12), 2044–2053. 21646122 10.3732/ajb.92.12.2044

[fsn31393-bib-0010] Nehdi, I. A. , Sbihi, H. , Tan, C. P. , & Al‐Resayes, S. I. (2013). Evaluation and characterisation of Citrullus colocynthis (L.) Schrad seed oil: Comparison with Helianthus annuus (sunflower) seed oil. Food Chemistry, 136(2), 348–353. 10.1016/j.foodchem.2012.09.009 23122069

[fsn31393-bib-0011] Nzeuwa, I. B. Y. , Belinda, I. , Guo, B. , Zhang, T. , Wang, L. , Ji, Q. , …, Sun, G. (2019). Comparative metabolic profiling of lycium fruits (Lycium barbarum and Lycium chinense) from different areas in China and from Nepal. Journal of Food Quality.2019, 6.

[fsn31393-bib-0012] Peng, Q. , Lv, X. , Xu, Q. , Li, Y. , Huang, L. , & Du, Y. (2012). Isolation and structural characterization of the polysaccharide LRGP1 from Lycium ruthenicum. Carbohydrate Polymers, 90(1), 95–101. 10.1016/j.carbpol.2012.04.067 24751015

[fsn31393-bib-0013] Qian, D. , Zhao, Y. , Yang, G. , & Huang, L. (2017). Systematic review of chemical constituents in the genus Lycium (Solanaceae). Molecules, 22(6), E911 10.3390/molecules22060911 28629116 PMC6152755

[fsn31393-bib-0014] Seeram, N. P. (2008). Berry fruits: Compositional elements, biochemical activities, and the impact of their intake on human health, performance, and disease. Journal of Agricultural and Food Chemistry, 56(3), 627–629. 10.1021/jf071988k 18211023

[fsn31393-bib-0015] Wang, H. Q. , Li, J. , Tao, W. , Zhang, X. , Gao, X. , Yong, J. , … Duan, J. (2018). Lycium ruthenicum studies: Molecular biology, Phytochemistry and pharmacology. Food Chemistry, 240, 759–766. 10.1016/j.foodchem.2017.08.026 28946340

[fsn31393-bib-0016] Xin, G. , Zhu, F. , Du, B. , & Xu, B. (2017). Antioxidants distribution in pulp and seeds of black and red goji berries as affected by boiling processing. Journal of Food Quality. 2017, 1–8. 10.1155/2017/3145946

[fsn31393-bib-0017] Zhang, D. L. , & Hamauzu, Y. (2004). Phenolics, ascorbic acid, carotenoids and antioxidant activity of broccoli and their changes during conventional and microwave cooking. Food Chemistry, 88(4), 503–509. 10.1016/j.foodchem.2004.01.065

[fsn31393-bib-0018] Zhao, X. , Dong, B. , Li, P. , Wei, W. , Dang, J. , Liu, Z. … Yue, H. (2018). Fatty acid and phytosterol composition, and biological activities of Lycium ruthenicum Murr. seed oil. Journal of Food Science, 83(10):2448–2456. 30178878 10.1111/1750-3841.14328

[fsn31393-bib-0019] Zheng, J. , Ding, C. , Wang, L. , Li, G. , Shi, J. , Li, H. , … Suo, Y. (2011). Anthocyanins composition and antioxidant activity of wild Lycium ruthenicum Murr. from Qinghai‐Tibet. Plateau. Food Chemistry, 126(3), 859–865. 10.1016/j.foodchem.2010.11.052

